# Viral transmission and evolution dynamics of SARS-CoV-2 in shipboard quarantine

**DOI:** 10.2471/BLT.20.255752

**Published:** 2021-04-30

**Authors:** Ting-Yu Yeh, Gregory P Contreras

**Affiliations:** a Auxergen Inc., Columbus Center, 701 East Pratt Street, Baltimore, MD 21202, United States of America.

## Abstract

**Objective:**

To examine transmission and evolution of severe acute respiratory syndrome coronavirus 2 (SARS-CoV-2) in shipboard quarantine of the Diamond Princess cruise ship.

**Methods:**

We obtained the full SARS-CoV-2 genome sequences of 28 samples from the Global Initiative on Sharing All Influenza Data database. The samples were collected between 10 and 25 February 2020 and came for individuals who had been tested for SARS-CoV-2 during the quarantine on the cruise ship. These samples were later sequenced in either Japan or the United States of America. We analysed evolution dynamics of SARS-CoV-2 using computational tools of phylogenetics, natural selection pressure and genetic linkage.

**Findings:**

The SARS-CoV-2 outbreak in the cruise most likely originated from either a single person infected with a virus variant identical to the WIV04 isolates, or simultaneously with another primary case infected with a virus containing the 11083G > T mutation. We identified a total of 24 new viral mutations across 64.2% (18/28) of samples, and the virus evolved into at least five subgroups. Increased positive selection of SARS-CoV-2 were statistically significant during the quarantine (Tajima’s *D*: −2.03, *P* < 0.01; Fu and Li’s *D*: −2.66, *P* < 0.01; and Zeng’s *E*: −2.37, *P* < 0.01). Linkage disequilibrium analysis confirmed that ribonucleic acid (RNA) recombination with the11083G > T mutation also contributed to the increase of mutations among the viral progeny.

**Conclusion:**

The findings indicate that the 11083G > T mutation of SARS-CoV-2 spread during shipboard quarantine and arose through *de novo* RNA recombination under positive selection pressure.

## Résumé

**Objectif:**

Étudier la transmission et l'évolution du coronavirus 2 du syndrome respiratoire aigu sévère (SARS-CoV-2) lors d'une quarantaine à bord du navire de croisière Diamond Princess.

**Méthodes:**

Nous avons obtenu l'ensemble des séquences génétiques du SARS-CoV-2 de 28 échantillons issus de la base de données de l'Initiative mondiale de partage des données sur la grippe aviaire (GISAID). Ces échantillons ont été collectés entre le 10 et le 25 février 2020 et prélevés sur des individus soumis à un test de dépistage du SARS-CoV-2 durant la quarantaine à bord du navire. Ils ont ensuite été séquencés soit au Japon, soit aux États-Unis d'Amérique. Nous avons analysé la dynamique d'évolution du SARS-CoV-2 à l'aide d'outils informatiques de phylogénétique, de pression sélective naturelle et de liaison génétique.

**Résultats:**

L'épidémie de SARS-CoV-2 à bord provient vraisemblablement d'une seule personne infectée par un variant identique aux isolats WIV04, ou simultanément d'un autre cas primaire d'infection par un virus contenant la mutation 11083G > T. Nous avons détecté au total 24 nouvelles mutations virales dans 64,2% (18/28) des échantillons, et le virus a évolué en cinq sous-groupes minimum. La sélection positive accrue de SARS-CoV-2 s'est révélée statistiquement significative durant la quarantaine (*D* de Tajima: −2,03, *P* < 0,01; *D *de Fu et Li: −2,66, *P* < 0,01; et *E *de Zeng: −2,37, *P* < 0,01). L'analyse du déséquilibre de liaison a confirmé que la recombinaison de l'acide ribonucléique (ARN) avec la mutation 11083G > T avait également contribué à la hausse des mutations dans la lignée virale.

**Conclusion:**

Ces résultats indiquent que la mutation 11083G > T du SARS-CoV-2 s'est répandue durant la quarantaine à bord du navire, et par le biais d'une recombinaison *de novo* de l'ARN sous une pression sélective positive.

## Resumen

**Objetivo:**

Examinar la transmisión y la evolución del coronavirus del síndrome respiratorio agudo grave 2 (SARS-CoV-2) en la cuarentena del crucero Diamond Princess.

**Métodos:**

Obtuvimos las secuencias completas del genoma del SARS-CoV-2 de 28 muestras de la base de datos de la Global Initiative on Sharing All Influenza Data. Las muestras se recogieron entre el 10 y el 25 de febrero de 2020 y procedían de individuos a los que se les había hecho la prueba del SARS-CoV-2 durante la cuarentena en el crucero. Estas muestras se secuenciaron posteriormente en Japón o en los Estados Unidos de América. Se analizó la dinámica evolutiva del SARS-CoV-2 utilizando herramientas computacionales de filogenética, presión de selección natural y enlace genético.

**Resultados:**

Lo más probable es que el brote de SARS-CoV-2 en el crucero se haya originado a partir de una sola persona infectada con una variante del virus idéntica a las cepas WIV04, o bien simultáneamente con otro caso primario infectado con un virus que contenía la mutación 11083G > T. Identificamos un total de 24 nuevas mutaciones virales en el 64,2% (18/28) de las muestras y el virus evolucionó en al menos cinco subgrupos. El aumento de la selección positiva del SARS-CoV-2 fue estadísticamente significativo durante la cuarentena (D de Tajima -2,03, P < 0,01; D de Fu y Li: -2,66, p < 0,01; y E de Zeng: -2,37, P < 0,01). El análisis de desequilibrio de linaje confirmó que la recombinación del ácido ribonucleico (ARN) con la mutación 11083G > T también contribuyó al aumento de las mutaciones entre la progenie viral.

**Conclusión:**

Los resultados indican que la mutación 11083G > T del SARS-CoV-2 se propagó durante la cuarentena en el crucero y surgió por recombinación de novo del ARN bajo presión de selección positiva.

## ملخص

الغرض التحقق من انتقال وتطور فيروس كورونا 2 التنفسي الحاد (سارس كوف 2) في الحجر الصحي على متن السفينة السياحية Diamond Princess.

الطريقة حصلنا على تسلسلات جينوم سارس كوف 2 الكامل من 28 عينة من المبادرة العالمية لمشاركة كل قاعدة بيانات الأنفلونزا. تم جمع العينات بين 10 و25 فبراير/شباط 2020، وجاءت من الأفراد الذين تم فحصهم للتحقق من إصابتهم بفيروس سارس كوف 2 أثناء الحجر الصحي على السفينة السياحية. تم ترتيب هذه العينات لاحقًا إما في اليابان أو الولايات المتحدة الأمريكية. وقمنا بتحليل آليات تطور فيروس سارس كوف 2 باستخدام الأدوات الحاسوبية لعلم الجينات الوراثة، وضغط الانتقاء الطبيعي، والارتباط الجيني.

النتائج كان تفشي فيروس سارس كوف 2 في الرحلة البحرية قد نشأ إما من شخص واحد مصاب بمتغير فيروسي مماثل للحالات المعزولة من WIV04، أو في نفس الوقت مع حالة أولية أخرى مصابة بفيروس يحتوي على طفرة 11083G > T. حددنا إجمالي 24 طفرة فيروسية جديدة عبر 64.2% (18/28) من العينات، وتطوّر الفيروس إلى خمس مجموعات فرعية على الأقل. كانت الاختيار الإيجابي المتزايد لفيروس سارس كوف 2 ملموساً من الناحية الإحصائية خلال الحجر الصحي (Tajima’s *D *: -2.03، نسبة الاحتمال أقل من 0.01؛ Fu and Li’s *D *: -2.66، نسبة الاحتمال أقل من 0.01؛ Zeng’s *E * : 2.37-؛ نسبة الاحتمال أقل من 0.01). أكد تحليل اختلال التوازن الخطي أن إعادة اتحاد الحمض النووي الريبوزي (RNA)، مع مساهمة طفرة 11083G > T كذلك في زيادة الطفرات بين سلالة الفيروس.

الاستنتاج تشير النتائج إلى أن الطفرة 11083G > T لفيروس سارس كوف 2، انتشرت أثناء الحجر الصحي على متن السفينة، ونشأت من خلال إعادة تركيب الحمض النووي الريبوزي *de novo * (المتكرر) تحت ضغط الاختيار الإيجابي.

## 摘要

**目的:**

旨在探讨“钻石公主”号邮轮上严重急性呼吸综合征冠状病毒 2 型 (SARS-CoV-2) 的传播和演变。

**方法:**

我们从全球共享流感数据倡议组织的数据库里获取了 28 份 SARS-CoV-2 样本的全基因组序列。这些样本的采集时间介于 2020 年 2 月 10 日至 25 日期间，采集对象是在邮轮隔离期间接受 SARS-CoV-2 检测的船上人员。然后，我们在美国或日本对这些样本进行了测序。我们利用种系遗传学、自然选择压力和遗传连锁相关计算工具分析了 SARS-CoV-2 的演变情况。

**结果:**

邮轮上之所以爆发 SARS-CoV-2 疫情，很有可能是因为有一个人感染了与 WIV04 分离毒株同源的变异病毒，或者另外还存在一位感染了 11083G > T 突变病毒的原发病例。我们在 64.2% (18/28) 的样本中总共发现了 24 种新的突变病毒，而且这些病毒演变成了至少 5 个亚群。在隔离检疫期间选择更多 SARS-CoV-2 检测结果为阳性的病例具有统计学意义（Tajima’s *D*：-2.03, *P* < 0.01；Fu and Li’s *D*：-2.66, *P* < 0.01；以及 Zeng’s *E*：-2.37, *P* < 0.01）。连锁不平衡分析证实了核糖核酸 (RNA) 重组与 11083G > T 突变也会提高病毒子代间的突变几率。

**结论:**

研究结果显示，SARS-CoV-2 病毒发生 11083G > T 突变传播的原因在于在船舶检疫隔离期间正性选择压力下 RNA 重组的新发突变。

## Резюме

**Цель:**

Изучить пути передачи и развитие коронавируса тяжелого острого респираторного синдрома коронавируса‑2 (SARS-CoV-2) в условиях карантина на борту круизного судна Diamond Princess.

**Методы:**

Авторы получили полные геномные последовательности SARS-CoV-2 из 28 образцов из базы данных Глобальной инициативы по обмену всеми данными о гриппе. Образцы были собраны в период с 10 по 25 февраля 2020 года у лиц, прошедших тест на SARS-CoV-2 во время карантина на круизном судне. Позже эти образцы секвенировали в Японии или Соединенных Штатах Америки. Авторы проанализировали динамику развития SARS-CoV-2, используя вычислительные инструменты филогенетики, давления отбора и генетического сцепления.

**Результаты:**

Скорее всего, вспышка SARS-CoV-2 во время круиза возникла либо от одного человека, инфицированного вариантом вируса, идентичным изолятам WIV04, либо одновременно с другим первичным случаем инфицирования вирусом, содержащим мутацию 11083G > T. Авторы идентифицировали в общей сложности 24 новые вирусные мутации в 64,2% (18/28) образцов, и вирус эволюционировал как минимум в пять подгрупп. Повышенный положительный отбор SARS-CoV-2 был статистически значимым во время карантина (значение теста *D* Таджимы: -2,03, *P* < 0,01; значение теста *D* Фу и Ли: -2,66, *P* < 0,01; значение теста *E* Цзэн: -2,37, *P* < 0,01). Анализ неравновесного сцепления генов подтвердил, что рекомбинация рибонуклеиновой кислоты (РНК) с мутацией 11083G > T также способствовала увеличению количества мутаций среди вирусного потомства.

**Вывод:**

Полученные результаты показывают, что мутация 11083G > T SARS-CoV-2 распространилась во время карантина на борту судна и возникла в результате *de novo* рекомбинации РНК под давлением положительного отбора.

## Introduction

On 31 December 2019, Chinese authorities alerted the World Health Organization (WHO) of an outbreak of a novel coronavirus causing severe illness with pneumonia-like symptoms. The virus was later named severe acute respiratory syndrome coronavirus 2 (SARS-CoV-2).^1^ WHO declared the outbreak a Public Health Emergency of International Concern on 30 January 2020^2^ and as of 17 December 2020, more than 76 million confirmed cases had been reported and 1.6 million people had died.^3^

SARS-CoV-2 contains a single positive stranded RNA (ribonucleic acid) of 30 kilobases, which encodes for 10 genes,^4^ and like other RNA viruses, evolves through random nucleotide substitutions. Early sequencing of samples from coronavirus disease 2019 (COVID-19) patients on 30 January 2020 showed only six mutations compared with the first isolate WIV04, and hence suggested that the virus had a short infection history in humans.^4^ By April 2020, researchers had found more than 131 mutations in SARS-CoV-2 across the 103 sequenced viral genomes and they estimated that the virus accumulates about one to two mutations per month.^5^

On 25 January 2020, a passenger disembarked the Diamond Princess cruise ship in Hong Kong Special Administrative Region, China, and on 1 February, the passenger tested positive for SARS-CoV-2. When the ship docked in Yokohama, Japan, on February 3, the 3711 cruise passengers and crew members had to quarantine on the ship. As of 21 February 2020, 712 (19.2%) of these individuals had tested positive for SARS-CoV-2, of which 331 (46.5%) were asymptomatic at the time of testing. Among the 381 symptomatic individuals, 37 (9.7%) required intensive care and nine (2.4%) died.^6^^,^^7^

The shipboard quarantine provided a closed environment to observe the SARS-CoV-2 transmission and adaptation independently from other infectious resources.^6^^,^^7^ This environment presents an ideal static population, with little interfering noise, to measure the viral phylodynamics from the COVID-19 outbreak. We therefore decided to use this opportunity to study *de novo* evolution of SARS-CoV-2 in a closed population.

## Methods

### Data resources

Viral sequences and sequencing methods are available in the Global Initiative on Sharing All Influenza Data (GISAID)^8^ and GenBank® databases.^9^ From these databases, we downloaded sequences and annotations of the isolates from the cruise ship, as well as the reference genomes of SARS-CoV-2 isolates PBCAMS-WH-04 (accession no. MT019532), WIV04 (MN996528), Hu-1 (NC045512) and WHU01 (MN98868).^4^

### Statistical and phylogenetics analyses

We aligned FASTA files of viral sequences using MAFFT 7 software (Kazutaka Katoh, Research Institute for Microbial Diseases, Osaka, Japan).^10^^,^^11^ To analyse phylogenetic relationships between viral sequences, we used the neighbour-joining method and Jukes–Cantor substitution model with setting bootstrap resampling number as five. We generated the rectangular phylogenetic tree using Archaeopteryx with Java plug-in of MAFFT 7.^12^ For radial phylogenetic tree, we first exported the tree file as Newick format by MAFFT; the FigTree software (version 1.4.2, Andrew Rambaut, University of Edinburgh, Edinburgh, United Kingdom of Great Britain and Northern Ireland) was used to transform and display the cladogram.^13^ To illustrate the viral subgroups with 11803G > T mutation, we rooted an unrooted tree by introducing the bat SARS-like CoV WIV16 (accession no. KT444582) as an outgroup virus.

#### Selection pressure

To determine whether the viral genome undergoes neutral or non-neutral evolution, that is, genetic variations of viral genomes are due to randomly genetic drift or under natural selection pressure, we used MEGA7 software^14^ to calculate Tajima’s *D* test of neutrality.^15^ This method compares the number of mutations per site with the nucleotide diversity (the mean pairwise difference between sequences). To compare the number of derived singleton site mutations – that is, single base mutations that occur only once in a given population – and the mean pairwise difference between sequences, we calculated Fu and Li’s *D*, using online PopSc calculator (Shi-Yi Chen, Sichuan Agricultural University, Chengdu, China). We used the same calculator for calculating Zeng’s *E*, which measures changes in high-frequency variants.^16^^–^^19^ To calculate the *P* values for Tajima’s *D*, Fu and Li’s *D* and Zeng’s *E* values, we used DnaSP 6 software (Julio Rozas, University of Barcelona, Barcelona, Spain).^20^

To investigate the linkage disequilibrium, that is the non-random assortment of alleles at different loci, of SARS-CoV-2 genomes, we first converted 148 SARS-CoV-2 genomic sequences using SNP_tools plug-in in Excel (Microsoft, Redmond, United States of America, USA) to create a baseline.^21^ We downloaded these sequences from GISAID. Using HaploView software, version 4.1 (Broad Institute, Cambridge, USA),^22^ we measured and plotted the normalized values (*D’*) of the coefficient of linkage disequilibrium (*D*). We obtained *D’* by dividing *D* with *D_max_*, where *D_max_* is the theoretical maximum difference between the observed and expected haplotype frequencies. We also calculated the log of the odds of there being a disequilibrium between two loci and the squared coefficient of correlation (*r^2^*) using the same software. In the absence of evolutionary forces or natural selection, the *D’* converges to zero along the time axis at a rate depending on the magnitude of the recombination rate between the two loci. We used the *χ^2^* test to examine if the obtained linkage disequilibrium was statistically significant. To detect positive RNA recombination, we plotted 95% confidence bounds for *D’* using HaploView.^23^ Pairs are thought to be in strong linkage disequilibrium if the upper 95% confidence bound is above 0.98 (that is, consistent with no recombination) and the lower bound is above 0.7. Conversely, strong evidence for recombination is defined if pairs for which the upper confidence bound of *D’* is less than 0.9. We searched a solid spine of strong linkage disequilibrium running from one marker to another along the legs of the triangle in the linkage disequilibrium chart to determine the haplotype block.^22^

## Results

### Viral variants

**Fig. 1:**
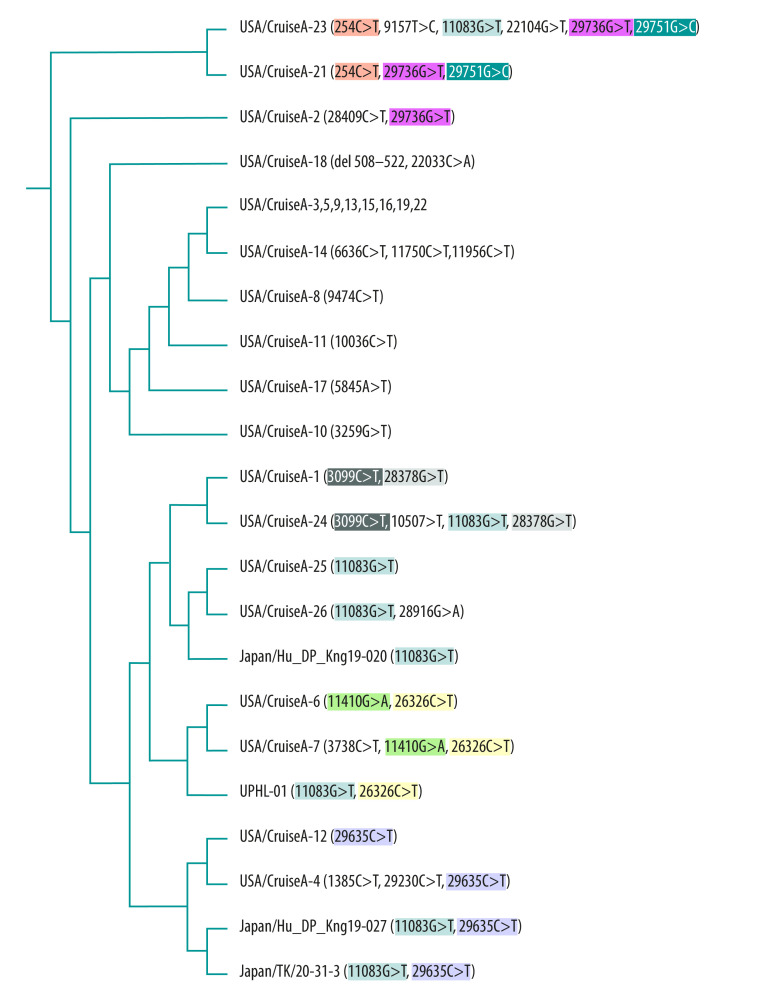
Phylogenetic tree of SARS-CoV-2 genomes from samples taken from people on shipboard quarantine, February 10 to February 25, 2020

A total of 28 specimens with viral sequences were available for this analysis, including 25 samples from the United States and three samples from Japan. Table 1 lists the characteristics of the viral sequences. Genetic variations of viral sequences were present in 71.4% (20/28) of samples. The sequences of eight samples were completely identical to the Wuhan isolates PBCAMS-WH-04, WIV04, Hu-1 and WHU01.^4^ A total of 24 new substitution mutations were identified in 18 samples (Table 1 and Fig. 1).

**Table 1:** Characteristics of SARS-CoV-2 genomes from samples taken from people on shipboard quarantine, February 10 to February 25, 2020

GISAID name	Accession no.	Specimen source	Collection date	Genomic change^a^	Type of mutation	Gene/protein	Amino acid change
hCoV-19/USA/CruiseA-1/2020	EPI_ISL_413606	Nasopharyngeal swab	17 Feb	3099C > T	Missense	*Orf1ab*/NSP3	T945I
				28378G > T	Synonymous	*N*	NA
hCoV-19/USA/CruiseA-2/2020	EPI_ISL_413607	Nasopharyngeal swab	18 Feb	28409C > T	Missense	*N*	P46S
				29736C > T	Non-coding	*3′-UTR*	NA
hCoV-19/USA/CruiseA-3/2020	EPI_ISL_413608	Nasopharyngeal swab	18 Feb	No change	NA	NA	NA
hCoV-19/USA/CruiseA-4/2020	EPI_ISL_413609	Nasopharyngeal swab	21 Feb	1385C > T	Synonymous	*Orf1ab*/NSP2	NA
				29230C > T	Synonymous	*N*	NA
				29635C > T	Non-coding	*3′-UTR*	NA
hCoV-19/USA/CruiseA-5/2020	EPI_ISL_413610	Oropharyngeal swab	21 Feb	No change	NA	NA	NA
hCoV-19/USA/CruiseA-6/2020	EPI_ISL_413611	Nasopharyngeal swab	21 Feb	11410G > A	Synonymous	*Orf1ab*/NSP6	NA
				26326C > T	Synonymous	*E*	NA
hCoV-19/USA/CruiseA-7/2020	EPI_ISL_413612	Nasopharyngeal swab	17 Feb	3738C > T	Missense	*Orf1ab*/NSP3	P1158S
				11410G > A	Synonymous	*Orf1ab*/NSP6	NA
				26326C > T	Synonymous	*E*	NA
hCoV-19/USA/CruiseA-8/2020	EPI_ISL_413613	Nasopharyngeal swab	17 Feb	9474C > T	Missense	*Orf1ab*/NSP4	A3070V
hCoV-19/USA/CruiseA-9/2020	EPI_ISL_413614	Nasopharyngeal swab	17 Feb	No change	NA	NA	NA
hCoV-19/USA/CruiseA-10/2020	EPI_ISL_413615	Nasopharyngeal swab	17 Feb	3259G > T	Missense	*Orf1ab*/NSP3	Q998H
hCoV-19/USA/CruiseA-11/2020	EPI_ISL_413616	Nasopharyngeal swab	17 Feb	10036C > T	Synonymous	*Orf1ab*/NSP4	NA
hCoV-19/USA/CruiseA-12/2020	EPI_ISL_413617	Oropharyngeal swab	20 Feb	29635C > T	Non-coding	*3′-UTR*	NA
hCoV-19/USA/CruiseA-13/2020	EPI_ISL_413618	Nasopharyngeal swab	20 Feb	No change	NA	NA	NA
hCoV-19/USA/CruiseA-14/2020	EPI_ISL_413619	Oropharyngeal swab	25 Feb	6636C > T	Missense	*Orf1ab*/NSP3	T2124I
				11750C > T	Missense	*Orf1ab*/NSP6	L3829F
				11956C > T	Synonymous	*Orf1ab*/NSP7	NA
hCoV-19/USA/CruiseA-15/2020	EPI_ISL_413620	Nasopharyngeal swab	18 Feb	No change	NA	NA	NA
hCoV-19/USA/CruiseA-16/2020	EPI_ISL_413621	Nasopharyngeal swab	18 Feb	No change	NA	NA	NA
hCoV-19/USA/CruiseA-17/2020	EPI_ISL_413622	Nasopharyngeal swab	24 Feb	5845A > T	Missense	*Orf1ab*/NSP3	K1860N
hCoV-19/USA/CruiseA-18/2020	EPI_ISL_413623	Nasopharyngeal swab	24 Feb	508_522del	Deletion	*Orf1ab*/NSP1	Deletion 82–86
				22033C > A	Missense	*S*	F157L
hCoV-19/USA/CruiseA-19/2020	EPI_ISL_414479	Nasopharyngeal swab	18 Feb	No change	NA	NA	NA
hCoV-19/USA/CruiseA-21/2020	EPI_ISL_414480	Oropharyngeal swab	21 Feb	254C > T	Non-coding	*5′-UTR*	NA
				29726G > T	Non-coding	*3′-UTR*	NA
				29751G > C	Non-coding	*3′-UTR*	NA
hCoV-19/USA/CruiseA-22/2020	EPI_ISL_414481	Nasopharyngeal swab	21 Feb	No change	NA	NA	NA
hCoV-19/USA/CruiseA-23/2020	EPI_ISL_414482	Nasopharyngeal swab	18 Feb	254C > T	Non-coding	*5′-UTR*	NA
				9157T > C	Synonymous	*Orf1ab*/NSP4	NA
				11083G > T	Missense	*Orf1ab*/NSP6	L3606F
				22104G > T	Missense	*S*	G181V
				29736G > T	Non-coding	*3′-UTR*	NA
				29751G > C	Non-coding	*3′-UTR*	NA
hCoV-19/USA/CruiseA-24/2020	EPI_ISL_414483	Oropharyngeal swab	17 Feb	3099C > T	Missense	*Orf1ab*/NSP3	T945I
				10507C > T	Synonymous	*Orf1ab*/NSP5	NA
				11083G > T	Missense	*Orf1ab*/NSP6	L3606F
				28378G > T	Synonymous	*N*	NA
hCoV-19/USA/CruiseA-25/2020	EPI_ISL_414484	Nasopharyngeal swab	17 Feb	11083G > T	Missense	*Orf1ab*/NSP6	L3606F
hCoV-19/USA/CruiseA-26/2020	EPI_ISL_414485	Oropharyngeal swab	24 Feb	11083G > T	Missense	*Orf1ab*/NSP6	L3606F
				28916G > A	Missense	*N*	G215S
hCoV-19/Japan/TK/20–31–3/2020	EPI_ISL_413459	Bronchial and lung autopsy	20 Feb	11083G > T	Missense	*Orf1ab*/NSP6	L3606F
				29635C > T	Non-coding	*3′-UTR*	NA
hCoV-19/Japan/Hu_DP_Kng_19–027/2020	EPI_ISL_412969	Throat swab	10 Feb	11083G > T	Missense	*Orf1ab*/NSP6	L3606F
				2963C > T	Non-coding	*3′-UTR*	NA
hCoV-19/Japan/Hu_DP_Kng_19–020/2020	EPI_ISL_412968	Throat swab	10 Feb	11083G > T	Missense	*Orf1ab*/NSP6	L3606F

del: deletion; E: envelope protein; GISAID: Global Initiative on Sharing All Influenza Data; N: nucleocapsid phosphoprotein; NA: not applicable; ORF: open reading frame; S: spike glycoprotein; SARS-CoV-2: severe acute respiratory syndrome coronavirus 2; UTR: untranslated region.

^a^ We compared the genomes to SARS-CoV-2 sequence to WIV04 (accession no. MN996528).

Note: The genome sequences are available at Global Initiative on Sharing All Influenza Data.^8^

SARS-CoV-2: severe acute respiratory syndrome coronavirus 2.

Notes: We generated the phylogenetic tree of the viral sequences from the cruise in MAFFT^10^ using the neighbour-joining method. Mutations appearing in more than one variant are colour coded. We have included UPHL-01 (accession no. EPI_ISL_415539U) as it is the first sample with both 11083G > T and 26326C > T.

Two mutations had previously been identified in other SARS-Cov-2 isolates. First, the deletion in hCoV-19/USA/CruiseA-18/2020 has been detected in USA-CA6 (MT044258). However, the possibility that hCoV-19/USA/CruiseA-18/2020 contributed to a new mutation in the cruise samples can be dismissed because this deletion was absent in the other samples. Second, the nonsynonymous mutation 11083G > T was found in USA-AZ1 (EPI_ISL_406223) on 22 January. Later, this mutation was also present in WA3-UW1 (EPI_ISL_413025), NY-NYUMC1 (EPI_ISL_414639) and UPHL-01 (EPI_ISL_415539U).

### Viral origin and transmission

**Fig. 2:**
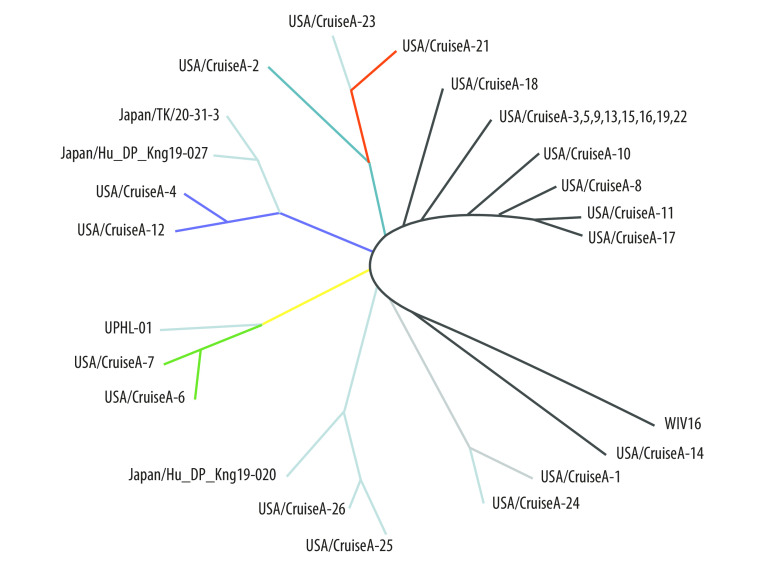
Rooted phylogenetic tree of SARS-CoV-2 genomes from samples taken from people on shipboard quarantine, February 10 to February 25, 2020

Fig. 1 shows the phylogenetic trees of the 28 samples without introducing the outgroup virus. Half (14/28) of the samples contained a virus variant that had evolved, that is, had more than two mutations, during the shipboard quarantine. We identified five subgroups after rooting the phylogenetic tree with an outgroup virus sequence of SARS-WIV16: (i) 3099C > T and 28378G > T (two samples); (ii) 11083G > T (three samples); (iii) 11410G > A and 26326C > T (two samples); (iv) 29635C > T (four samples); and (v) 29736G > T (three samples; Fig. 2). Due to 11083G > T mutation, the clustering of taxa on viral phylogenies was obvious with spatially structured host population between all these subgroups (50%; 14/28; Fig. 2). 

SARS-CoV-2: severe acute respiratory syndrome coronavirus 2.

Notes: Alignments of viral sequences were generated using MAFFT,^10^ and the phylogenetic tree was visualized using FigTree. Rooting was done by introducing bat SARS-like coronavirus WIV16 (accession no. KT444582) as an outgroup virus. Each colour represents a subgroup.

Whether the single 11083G > T substitution spontaneously occurred during the quarantine or the patients had been infected with a viral variant containing this mutation before boarding the ship is unclear. Nevertheless, all of the viral sequences were more similar to the WIV04 sequence than with other 143 SARS-CoV-2 isolates in the GISAID database (data repository).^24^ This result suggests that the 24 new mutations identified were generated *de novo* on the ship rather than deriving from multiple geographical origins.

The analysis revealed two possibilities of the viral origin: either the virus (except hCoV-19/USA/CruiseA-18/2020) originated from a single primary case with the WIV04 sequence and all substitution mutations occurred during the quarantine; or there were two simultaneously primary cases, one identical to the WIV04 sequence and one containing the 11083G > T substitution.

### Natural selection of mutations

Four variants had three mutations in their genome, one variant had four mutations and two variants had six mutations (Fig. 1 and Table 1). To test the hypothesis that the virus mutation evolved under selection pressure as opposed to neutral evolution (random) onboard the cruise ship, we calculated Tajima’s *D* to test neutrality of DNA polymorphisms.^15^^,^^25^ The Tajima’s *D* value in the cruise was −2.03 (*P* < 0.01) compared with 6.75 among 143 full-length genomic sequences of SARS-CoV-2 isolates sequenced between 10 January and 13 March 2020. This result indicates that, while other SARS-CoV-2 isolates faced balancing selection (due to a positive Tajima’s *D*), the virus spreading on the cruise had evolved under two possible but not exclusive forces generated during the quarantine process: purifying or positive selection; and population growth of the new virus variant among infected patients after a recent bottleneck caused by the quarantine.

We further investigated these two possibilities using two neutrality tests.^16^^,^^17^ First, Fu and Li’s test generated a negative *D* value of −2.66 (*P* < 0.01), suggesting that the quarantine procedure provided a purifying or positive selection pressure to generate an excess of singleton sites. This conclusion was corroborated by the fact that the 39.3% (11/28) of cases contained 15 new singleton mutations (Fig. 1). Second, Zeng’s *E* value of  −2.37 (*P* < 0.01) supported the possibility of population growth of the virus after a recent bottleneck as a force. We conclude that SARS-CoV-2 viral evolution was positively correlated to the increase of the selection pressure during the shipboard quarantine.

### RNA recombination

Seven samples contain the 11083G > T mutation although they belong to different subgroups (Fig. 1). By assuming the substitution rate of 0.92 × 10^−3^/site/year,^26^ it is unlikely that the virus variants of different subgroups all generated the same spontaneous mutation at the G11083 site into the nucleotide T within three weeks. One hypothesis is that RNA recombination occurred in these cases to gain the 11083G > T mutation.

**Fig. 3:**
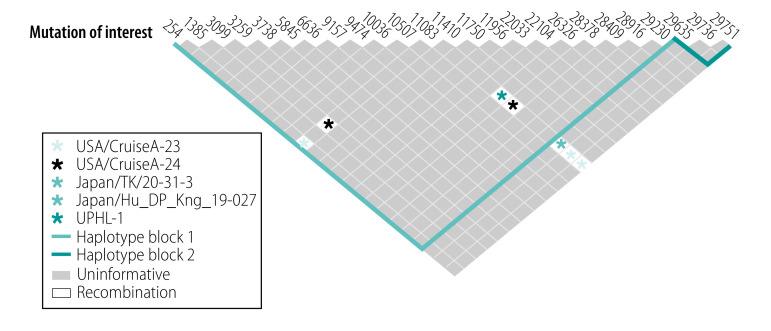
Haplotype block organization of SARS-CoV-2 mutations in samples from the cruise ship Diamond Princess, 2020

To determine whether four variants from different subgroups (samples /USA/CruiseA-23/, /USA/CruiseA-24/, /Japan/TK/20–31–3/2020 and /Japan/Hu_DP_Kng_19–027/2020) obtained the G11083 > T mutation via RNA recombination, we analysed the patterns of linkage disequilibrium between variants with minor alleles of two SARS-CoV-2 variants (available in the data repository).^24^ Table 2 shows five pairs of mutations among all analysed samples with high log of the odds of there being a disequilibrium between two loci (> 3.0) and their *r*^2^ values. The linkages between double mutations in variants /USA/CruiseA-1/ and /USA/CruiseA-24/, /USA/CruiseA-6/ and /USA/CruiseA-7/, and /USA/CruiseA-21/ and /USA/CruiseA-23/ were statistically significant (*P* < 0.001). Furthermore, there was indication of positive RNA recombination. The upper 95% confidence bound of *D’* for C254/G11083 was 0.86, for G29736/G11083 was 0.73, for G29751/G11083 was 0.86, for C3099/G11083 was 0.86, for G28378/G11083 was 0.86 and for C29635/G11083 was 0.80 (Fig. 3). This result supports the hypothesis that 11083G > T mutation had been gained via RNA recombination in these four variants. 

**Table 2:** Linkage disequilibrium of SARS-CoV-2 mutations with high log of the odds and the *r*^2^ values

Pair	Log of the odds	*r*^2^
C3099/G28378	4.60	1.00
G11410/C26326	3.77	0.66
G29736/29751	3.77	0.66
C254/G29736	3.77	0.66
C254/G29751	4.60	1.00

SARS-CoV-2: severe acute respiratory syndrome coronavirus 2.

Notes: Linkage disequilibrium plot of HaploView to display the confidence bounds colour scheme. Each box represents a pair of mutations. The solid spines of strong linkage disequilibrium running from one marker to another along the legs of the triangle in the linkage disequilibrium chart determine the haplotype block.^22^ We defined strong evidence of recombination if pairs for which the upper confidence bound of the coefficient of linkage disequilibrium is less than 0.9 (white squares). We did not detect any strong linkage.

We detected the mutations 11083G > T and 26326 C > T, present in the UPHL-01 sequence, in the two variants: /USA/CruiseA-6/ and /USA/CruiseA-7/. The upper 95% confidence bounds on *D’* of C26326/G11083 was 0.73 (Fig. 3), suggesting that a recent RNA recombination event may have also occurred in UPHL-01.

**Fig. 4:**
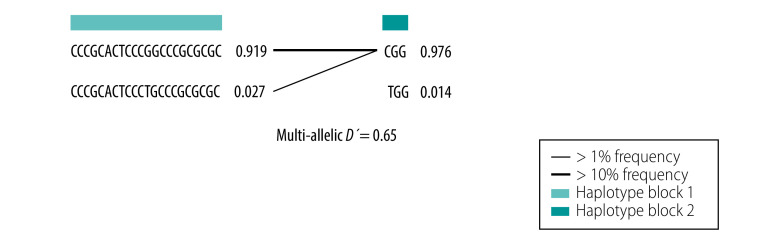
Haplotype frequencies of SARS-CoV-2 mutations in the cruise ship Diamond Princess, 2020

Fig. 4 shows each haplotype in the two identified blocks and their population frequencies and connections between blocks. The value of multi-allelic *D'* was 0.65, which represented the level of recombination between the two blocks. These data provide evidence that RNA recombination contributed to SARS-CoV-2 mutations during shipboard quarantine.

SARS-CoV-2: severe acute respiratory syndrome coronavirus 2.

Notes: Numbers next to each haplotype block are haplotype frequencies. The joining lines represent combined haplotypes. In the crossing areas between haplotype blocks, a value of multi-allelic *D*’, that is the normalized value of the coefficient of linkage disequilibrium, is shown to represent the level of recombination between blocks.

### Protein mutations

The viral populations in the cruise also generated 11 new variants in the viral proteins, including proteins in the gene *ORF1ab* (nonstructural protein NSP3, NSP4, NSP6), and in the viral structural spike and nucleocapsid proteins (Table 1). We found five missense variants in NSP3: T945I; Q998H; P1158S in the macro domain; K1860N in papain-like protease; and T2124I in the group 2 marker domain. In the other NSPs, we detected two mutations: A3070V in NSP4 and L3829F in NSP6. For the spike protein, two mutations, F157L and G181V, were identified. For the nucleocapsid phosphoprotein, we found mutations in the RNA binding domain (P46S) and the arginine-serine domain (G215S).

Only variant /USA/CruiseA-14/ had two mutations, T2124I and L3829F in NSP3 and NSP6, respectively (Table 1).

### 3’-UTR mutations

**Fig. 5:**
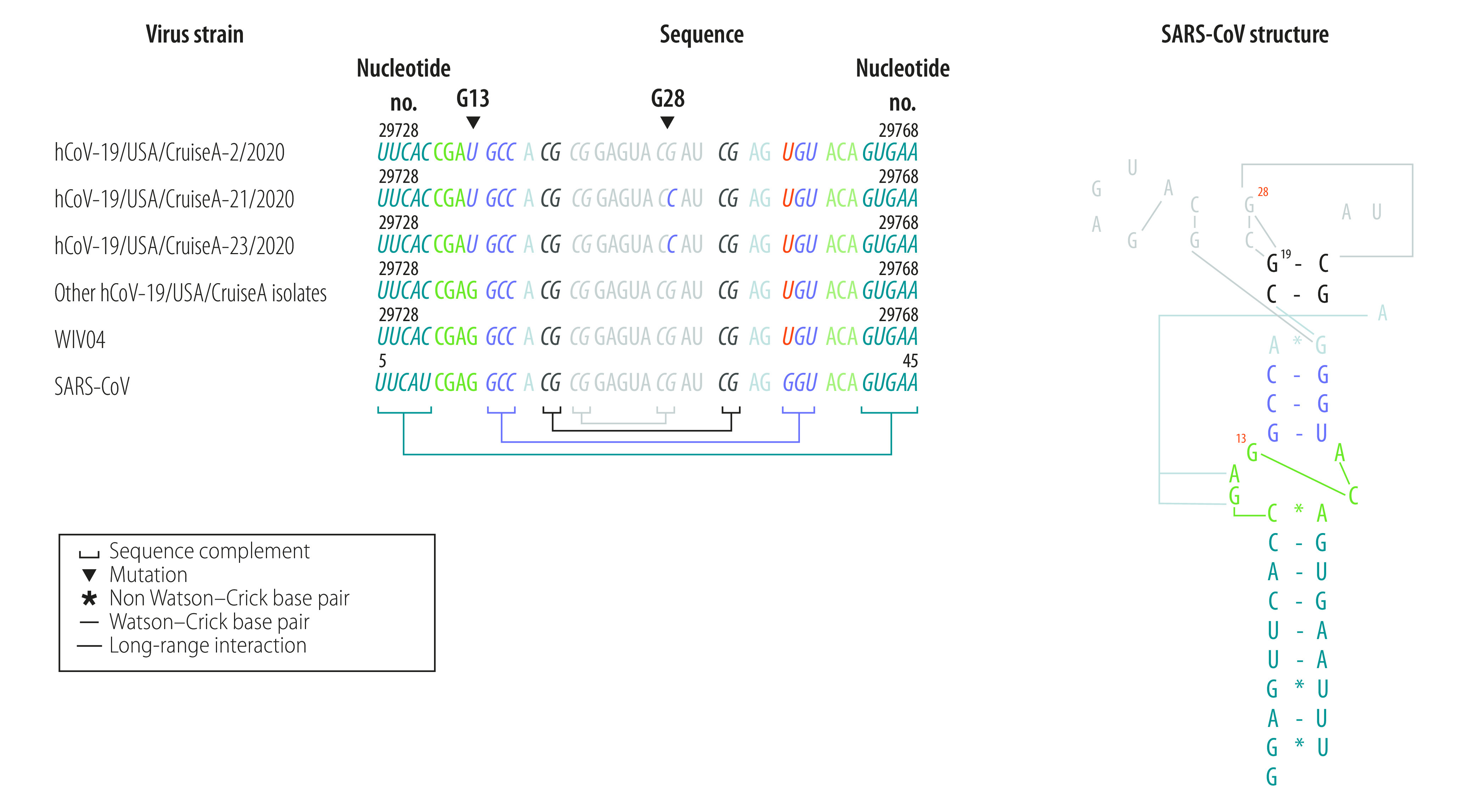
Mutations in the SARS-CoV-2 stem loop-II RNA motif

In three samples, we found two mutations, 29736G > T and 29751G > T in the stem loop-II motif (Fig. 5). We used the published three-dimensional crystal structure of SARS-CoV stem loop-II motif RNA^27^ to map the nucleotides G29736 and G29751. We found that these nucleotides are equal to the nucleotides G13 and G28 in the SARS-CoV stem loop-II motif (Fig. 5). In SARS-CoV, G13 (G29736 in SARS-CoV-2) forms a base triple with A38 and C39 in a seven-nucleotide asymmetric bubble, while G28 (G29751 in SARS-CoV-2) participates in formation of an essential RNA base quartet composed of two G–C pairs (G19, C20, G28, C31; Fig. 5).^29^

SARS-CoV: severe acute respiratory syndrome coronavirus.

Notes: We compared hCoV-19/USA/CruiseA-2/2020 (accession no. EPI_ISL_413607), hCoV-19/USA/CruiseA-21/2020 (EPI_ISL_414480), hCoV-19/USA/CruiseA-23/2020 (EPI_ISL_414482) and other cruise isolates with WIV04 (MN996528) and SARS-CoV (NC004718). Conventional RNA helical base-pairing are indicated in italics. The schematic representation of the stem loop-II RNA secondary structure of SARS-CoV virus shows nucleotide interactions.^27^ The digits 13 and 28 show the location of the mutations identified in some of the cruise ship isolates and digit 19 represents the location of stem loop-II mutation found in Australia.^28^

## Discussion

Viral phylogenetics is a useful tool to study epidemiological and evolutionary processes, such as epidemic spread and spatiotemporal dynamics including metapopulation dynamics, zoonotic transmission, tissue tropism and antigenic drift.^30^ Here we report the viral phylodynamics of SARS-CoV-2 from patients in a shipboard quarantine for three weeks in February 2020. The transmission started from either one or two primary cases with WIV04 sequence and/or 11083G > T mutation, then quickly separated into at least five subgroups based on new mutations. Increased positive selection as well as RNA recombination of SARS-CoV-2 were evident during the quarantine. These results should be considered in formulation of future management protocols with respect to a SARS-CoV-2 outbreak in any relatively close quarters, such as shipboards, submarines, dormitories, prisons and hospitals.

While the quarantine averted a lot of infections on the shipboard,^31^ the phylogenetics analysis showed that viral transmission and RNA recombination occurred between the five identified subgroups. Our data fit in the coalescent model, which uses the diversity of viral genome, the viral evolutionary rate and the estimated time of infection to determine the number of viral genotypes present in the initial infected population.^32^ However, we cannot rule out that evolutionary processes, such as the transmission bottleneck that determines how much of the viral diversity generated in one host passes to another during transmission, also shaped the viral phylogenies. While spatial structure is the most general virus population structure in phylodynamic analyses, SARS-CoV-2 evolution may also have been influenced by the characteristic of the host, such as age, race and risk behaviour.^30^ Because viral transmission can preferentially occur between patients sharing any of these attributes, the real reason(s) for viral transmission between virus variants require(s) further study. Furthermore, studies on whether quarantine in close quarters also promotes virus to rapidly gain more mutations via RNA recombination are needed.

Despite the small sample size in this study, our findings from computational and statistical analyses indicated that the selection pressure was not random. We assume that SARS-CoV-2 variants were at an initial stage of evolution rather than the fixation stage,^33^ since the COVID-19 outbreak had only started 8 weeks before the Diamond Princess incident. The tools we used to measure selection pressure of adaptive evolution were Tajima’s *D,* Zeng’s *E* and Fu and Li’s *D* tests, because of their appropriateness in relation to the sample characteristics. For Tajima’s *D,* its power is not affected by RNA recombination events, number of segregation sites and various timescales nor does this test require the outgroup sequence or large sample size.^25^ Fu and Li’s *D* test has similar properties, but is more sensitive when singleton sites are in excess, as in our samples. As the 28 specimens were collected on the cruise ship and sequenced in Japan and the USA, the hypothesis of strict neutrality may not apply. In this case, Li et al.’s methods could improve the neutrality test for sequence polymorphisms from multiple samples.^34^ Unfortunately, we could not apply this method, since the detailed parameters needed for this method were not available in GISAID. Zeng’s *E* test has two advantages. First, this test is not affected by positive selection at various evolution stages but denotes powerful evidence for virus population growth. Second, the test is almost absent of any power to detect positive selection at this early stage of virus transmission, therefore the negative value is mainly contributed by the virus population growth.^17^ We excluded the method to calculate the ratio of nucleotide substitution rates, denoted dN/dS, despite the simplicity and robustness of this method. The reasons for this exclusion were: (i) the ratio is defined to describe the relative rate of selected versus neutral fixation events over long timescales, not transient polymorphisms over short timescales; and (ii) dN/dS value in a single population does not follow a monotonic function in proportion to positive selection pressure like diverged sequences.^35^

From these analyses of selection pressure, we conclude that on the cruise, the virus evolved under strong positive selection or maybe in the process of selective sweeps, which could generate beneficial mutations for SARS-CoV-2 to quickly reach fixation. 

Although RNA recombination in SARS-CoV-2 had been suggested previously,^28^ this study provides evidence that RNA recombination occurred *de novo* in SARS-CoV-2 genome. Within 3 weeks, the genome, sampled from four infected individuals, had gained the same 11083G > T mutation, suggesting that RNA recombination also participated in viral evolution of the virus. The RNA recombination of 11803G > T is also present in UPHL-01 variant, however, whether the carrier of the UPHL-01 acquired the variant from a cruise ship passenger or if the mutation appeared independently of cruise ship variants is unknown. Because this mutation was also later detected in other variants,^36^ in addition to UPHL-01, future studies should further investigate whether 11083G > T may increase the fitness of the carrier. Other studies have suggested the 11083G > T could be a beneficial mutation linked to asymptomatic infection.^36^^,^^37^

Of the 24 mutations we identified, 11 mutations led to amino acid substitutions and two mutations occurred in the stem loop-II motif in the 3’-UTR region. This motif is a very well conserved RNA motif in more than 30 coronaviruses.^29^^,^^38^ We have also reported the unique 29742G > A or 29742G > U substitutions in stem loop-II motif RNA in SARS-CoV-2 isolates in Australia (Fig. 5),^28^ reinforcing the idea that stem loop-II motif is a hotspot for mutations in SARS-CoV-2 rather than a conserved RNA domain.^27^ Mutations in this motif may disrupt RNA structure and thereby alter the viral viability or infectivity. Whether the mutations identified here, both in viral proteins and regulatory RNA regions, may enhance adaptation or attenuate virus replication or virulence^39^ requires further investigation. 

We acknowledge there may be limitations in collection of patients’ samples and size in extending our findings to represent the entire SARS-CoV-2-positive population (712 people, from January 20 to March 8, 2020) in the Diamond Princess cruise.^6^ If possible, further investigation with more viral genome sequences is required to understand the detailed evolutionary lineage of transmission and whether there is difference of viral mutations between symptomatic and asymptomatic individuals.

## References

[1] Novel Coronavirus – China. Geneva: World Health Organization; 2020. Available from: https://www.who.int/csr/don/12-january-2020-novel-coronavirus-china/en/[cited 2020 Jan 12].

[2] Statement on the second meeting of the International Health Regulations (2005) Emergency Committee regarding the outbreak of novel coronavirus (2019-nCoV). Geneva: World Health Organization; 2020. Available from: https://www.who.int/news/item/30-01-2020-statement-on-the-second-meeting-of-the-international-health-regulations-(2005)-emergency-committee-regarding-the-outbreak-of-novel-coronavirus-(2019-ncov) [cited 2020 Jan 30].

[3] COVID-19 dashboard [internet]. Baltimore: Johns Hopkins University; 2021. Available from: https://coronavirus.jhu.edu/map.html [cited 2020 Dec 17].

[4] Lu R, Zhao X, Li J, Niu P, Yang B, Wu H, et al. Genomic characterisation and epidemiology of 2019 novel coronavirus: implications for virus origins and receptor binding. Lancet. 2020 2 22;395(10224):565–74. 10.1016/S0140-6736(20)30251-8 32007145 PMC7159086

[5] Tang X, Wu C, Li X, Song Y, Yao X, Wu X, et al. On the origin and continuing evolution of SARS-CoV-2. Natl Sci Rev. 2020 6 3;7(6):1012–102. 10.1093/nsr/nwaa036 PMC7107875 34676127

[6] Moriarty LF, Plucinski MM, Marston BJ, Kurbatova EV, Knust B, Murray EL, et al.; CDC Cruise Ship Response Team; California Department of Public Health COVID-19 Team; Solano County COVID-19 Team. Public health responses to COVID-19 outbreaks on cruise ships - Worldwide, February-March 2020. MMWR Morb Mortal Wkly Rep. 2020 3 27;69(12):347–52. 10.15585/mmwr.mm6912e3 32214086 PMC7725517

[7] Kakimoto K, Kamiya H, Yamagishi T, Matsui T, Suzuki M, Wakita T. Initial investigation of transmission of COVID-19 among crew members during quarantine of a cruise ship - Yokohama, Japan, February 2020. MMWR Morb Mortal Wkly Rep. 2020 3 20;69(11):312–13. 10.15585/mmwr.mm6911e2 32191689 PMC7739985

[8] Global Initiative on Sharing All Influenza Data [internet]. Munich: Freunde von GISAID e.V.; 2021. Available from: https://www.gisaid.org/ [cited 2021 Feb 5].

[9] GenBank overview [internet]. Bethesda: National Institute of Health; 2021. Available from: https://www.ncbi.nlm.nih.gov/genbank [cited 2021 Feb 5].

[10] Katoh K, Standley DM. MAFFT multiple sequence alignment software version 7: improvements in performance and usability. Mol Biol Evol. 2013 4;30(4):772–80. 10.1093/molbev/mst010 23329690 PMC3603318

[11] MAFFT version 7 [internet]. Osaka: Research Institute for Microbial Diseases; 2021. Available from: https://mafft.cbrc.jp/alignment/server/index.html [cited 2021 Feb 5].

[12] Kuraku S, Zmasek CM, Nishimura O, Katoh K. aLeaves facilitates on-demand exploration of metazoan gene family trees on MAFFT sequence alignment server with enhanced interactivity. Nucleic Acids Res. 2013 7;41(Web Server issue):W22-8. 10.1093/nar/gkt389 23677614 PMC3692103

[13] Rambaut A. FigTree, version 1.4.2 [internet]. San Fransisco: GitHub, Inc.; 2021. Available from: https://github.com/rambaut/figtree/releases [cited 2021 Feb 5].

[14] Kumar S, Stecher G, Tamura K. MEGA7: Molecular evolutionary genetics analysis Version 7.0 for bigger datasets. Mol Biol Evol. 2016 7;33(7):1870–4. 10.1093/molbev/msw054 27004904 PMC8210823

[15] Tajima F. Statistical method for testing the neutral mutation hypothesis by DNA polymorphism. Genetics. 1989 11;123(3):585–95. 10.1093/genetics/123.3.585 2513255 PMC1203831

[16] Fu YX, Li WH. Statistical tests of neutrality of mutations. Genetics. 1993 3;133(3):693–709. 10.1093/genetics/133.3.693 8454210 PMC1205353

[17] Zeng K, Fu YX, Shi S, Wu CI. Statistical tests for detecting positive selection by utilizing high-frequency variants. Genetics. 2006 11;174(3):1431–9. 10.1534/genetics.106.061432 16951063 PMC1667063

[18] Chen SY, Deng F, Huang Y, Li C, Liu L, Jia X, et al. PopSc: computing toolkit for basic statistics of molecular population genetics simultaneously implemented in web-based calculator, Python and R. PLoS One. 2016 10 28;11(10):e0165434. 10.1371/journal.pone.0165434 27792763 PMC5085088

[19] popsc 1.0.1 [internet]. Fredericksburg: Python Software Foundation 2021. Available from: https://pypi.org/project/popsc [cited 2021 Feb 5].

[20] Rozas J, Ferrer-Mata A, Sánchez-DelBarrio JC, Guirao-Rico S, Librado P, Ramos-Onsins SE, et al. DnaSP 6: DNA sequence polymorphism analysis of large data sets. Mol Biol Evol. 2017 12 1;34(12):3299–302. 10.1093/molbev/msx248 29029172

[21] Chen B, Wilkening S, Drechsel M, Hemminki K. SNP_tools: a compact tool package for analysis and conversion of genotype data for MS-Excel. BMC Res Notes. 2009 10 23;2(1):214. 10.1186/1756-0500-2-214 19852806 PMC2771038

[22] Barrett JC, Fry B, Maller J, Daly MJ. Haploview: analysis and visualization of LD and haplotype maps. Bioinformatics. 2005 1 15;21(2):263–5. 10.1093/bioinformatics/bth457 15297300

[23] Gabriel SB, Schaffner SF, Nguyen H, Moore JM, Roy J, Blumenstiel B, et al. The structure of haplotype blocks in the human genome. Science. 2002 6 21;296(5576):2225–9. 10.1126/science.1069424 12029063

[24] Yeh TY, Contreras GP. Supplemental file for viral transmission and evolution dynamics of SARS-CoV-2 in shipboard quarantine [data repository]. London: Figshare; 2021. 10.6084/m9.figshare.14472966 PMC8243027 34248221

[25] Ramírez-Soriano A, Ramos-Onsins SE, Rozas J, Calafell F, Navarro A. Statistical power analysis of neutrality tests under demographic expansions, contractions and bottlenecks with recombination. Genetics. 2008 5;179(1):555–67. 10.1534/genetics.107.083006 18493071 PMC2390632

[26] Fang B, Liu L, Yu X, Li X, Ye G, Xu J, et al. Genome-wide data inferring the evolution and population demography of the novel pneumonia coronavirus (SARS-CoV-2). bioaRxiv 2020 3 11. 10.1101/2020.03.04.976662

[27] Robertson MP, Igel H, Baertsch R, Haussler D, Ares M Jr, Scott WG. The structure of a rigorously conserved RNA element within the SARS virus genome. PLoS Biol. 2005 1;3(1):e5. 10.1371/journal.pbio.0030005 15630477 PMC539059

[28] Yeh TY, Contreras GP. Emerging viral mutants in Australia suggest RNA recombination event in the SARS-CoV-2 genome. Med J Aust. 2020 7;213(1):44–44.e1. 10.5694/mja2.50657 32506536 PMC7300921

[29] Tengs T, Jonassen CM. Distribution and evolutionary history of the mobile genetic element s2m in coronaviruses. Diseases. 2016 7 28;4(3):E27. 10.3390/diseases4030027 28933407 PMC5456283

[30] Volz EM, Koelle K, Bedford T. Viral phylodynamics. PLOS Comput Biol. 2013;9(3):e1002947. 10.1371/journal.pcbi.1002947 23555203 PMC3605911

[31] Mizumoto K, Chowell G. Transmission potential of the novel coronavirus (COVID-19) onboard the Diamond Princess cruises ship, 2020. Infect Dis Model. 2020 2 29;5:264–70. 10.1016/j.idm.2020.02.003 32190785 PMC7068636

[32] McCrone JT, Lauring AS. Genetic bottlenecks in intraspecies virus transmission. Curr Opin Virol. 2018 2;28:20–5. 10.1016/j.coviro.2017.10.008 29107838 PMC5835166

[33] Patwa Z, Wahl LM. The fixation probability of beneficial mutations. J R Soc Interface. 2008 11 6;5(28):1279–89. 10.1098/rsif.2008.0248 18664425 PMC2607448

[34] Li H, Zhang Y, Zhang YP, Fu YX. Neutrality tests using DNA polymorphism from multiple samples. Genetics. 2003 3;163(3):1147–51. 12663551 10.1093/genetics/163.3.1147 PMC1462489

[35] Kryazhimskiy S, Plotkin JB. The population genetics of dN/dS. PLoS Genet. 2008 12;4(12):e1000304. 10.1371/journal.pgen.1000304 19081788 PMC2596312

[36] Wang R, Chen J, Hozumi Y, Yin C, Wei GW. Decoding asymptomatic COVID-19 infection and transmission. J Phys Chem Lett. 2020 12 3;11(23):10007–15. 10.1021/acs.jpclett.0c02765 33179934 PMC8150094

[37] Alejandro Lopez-Rincon A, Tonda A, Mendoza-Maldonado L, Claassen E, Garssen J, Kraneveld AD. A Missense mutation in SARS-CoV-2 potentially differentiates between asymptomatic and symptomatic cases [preprint]. Bull World Health Organ. E-pub: 9 April 2020. 10.2471/BLT.20.258889 10.2471/BLT.20.258889

[38] Su S, Wong G, Shi W, Liu J, Lai ACK, Zhou J, et al. Epidemiology, genetic recombination, and pathogenesis of coronaviruses. Trends Microbiol. 2016 6;24(6):490–502. 10.1016/j.tim.2016.03.003 27012512 PMC7125511

[39] Muth D, Corman VM, Roth H, Binger T, Dijkman R, Gottula LT, et al. Attenuation of replication by a 29 nucleotide deletion in SARS-coronavirus acquired during the early stages of human-to-human transmission. Sci Rep. 2018 10 11;8(1):15177. 10.1038/s41598-018-33487-8 30310104 PMC6181990

